# Internal and External Factors Related to Burnout among Iron and Steel Workers: A Cross-Sectional Study in Anshan, China

**DOI:** 10.1371/journal.pone.0143159

**Published:** 2015-11-17

**Authors:** Haiqiang Guo, Huifang Guo, Yilong Yang, Baozhi Sun

**Affiliations:** 1 School of Public Health, Harbin Medical University, Harbin, P.R. China; 2 Department of Nursing, the First Affiliated Hospital of Dalian Medical University, Dalian, P.R. China; 3 School of Public Health, China Medical University, Shenyang, P.R. China; National Center of Neurology and Psychiatry, JAPAN

## Abstract

**Background:**

Burnout is a syndrome of emotional exhaustion, cynicism and reduced professional efficacy, which can result from long-term work stress. Although the burnout level is high among iron and steel workers, little is known concerning burnout among iron and steel worker. This study aimed to evaluate the burnout and to explore its associated internal and external factors in iron and steel workers.

**Methods:**

A cross-sectional survey was conducted in iron and steel workers at the Anshan iron-steel complex in Anshan, northeast China. Self-administered questionnaires were distributed to 1,600 workers, and finally 1,300 questionnaires were returned. Burnout was measured using the Chinese version of the Maslach Burnout Inventory-General Survey (MBI-GS). Effort-reward imbalance (ERI), perceived organizational support (POS), and psychological capital (PsyCap) were measured anonymously. A hierarchical regression model was applied to explore the internal and external factors associated with burnout.

**Results:**

Mean MBI-GS scores were 13.11±8.06 for emotional exhaustion, 6.64±6.44 for cynicism, and 28.96±10.39 for professional efficacy. Hierarchical linear regression analysis showed that ERI and POS were the most powerful predictors for emotional exhaustion and cynicism, and PsyCap was the most robust predictor for high professional efficacy.

**Conclusions:**

Chinese iron and steel workers have a high level of burnout. Burnout might be associated with internal and external factors, including ERI, POS, and PsyCap. Further studies are recommended to develop an integrated model including both internal and external factors, to reduce the level of ERI, and improve POS and workers’ PsyCap, thereby alleviating the level of burnout among iron and steel workers.

## Background

Burnout is a psychological term that refers to long-term exhaustion and diminished interest in work, and is often defined as a syndrome consisting of emotional exhaustion, cynicism, and reduced professional efficacy [1]. Burnout occurs in a wide variety of occupations [2–5], and several studies have found that burnout is associated with many symptoms and possible consequences such as decreased job performance, increased rates of turnover, decreased career productivity, depression, and substance abuse [2,6]. As a result, burnout is increasingly receiving attention in the field of occupational health worldwide [7,8]. However, most studies have mainly focused on burnout problems in people-oriented professions such as health care and social services [2,5,6,8], and little is known concerning occupational burnout in the iron and steel industry, where the frontline production workers are highly exposed to toxic substances that are associated with a high risk of burnout [9].

Iron and steel production workers carry out a number of tasks (e.g., iron making, steelmaking, and steel rolling) in iron and steel industries consisting of oven and furnaces, and thus highly exposed to many toxic substances such as metals (e.g., chromium, manganese, lead, and cadmium), carbon monoxide, various dusts, fumes, acid mists, solvents, oil mists, and physical hazards including heat, noise, ionizing radiation and vibration [9]. Therefore, iron and steel workers are highly associated with many health ailments related to the musculoskeletal system, gastrointestinal system, and respiratory system [10,11]. In addition, it has been reported that the hypertension prevalence rate is strikingly high in Chinese iron and steel workers [12]. More seriously, some studies have found that the incidence rates of cancer (e.g., stomach, lung, bladder and colorectal cancer) are very high among the workers [9,13–15]. Besides, the high rate of absenteeism and the low level of subjective well-being are also observed in this population [10,16]. Occupational burnout is considered to result from chronic work stress [7]. However, little is known regarding burnout and its related factors in iron and steel workers, though they constantly experience high levels of work stress. Furthermore, in China, workers may often have to face high job demands, low rewards, and insecure/unfair employment system [17]. The shocking events that Chinese steel workers killed their manager and clashed with the police partly indicate that steel workers experience psychological problems that exacerbate burnout [18,19]. Therefore, identification of the associated factors of burnout among Chinese iron and steel workers is important in developing interventions to reduce burnout and to increase job performance.

Occupational psychosocial factors associated with burnout are roughly divided into internal (e.g., personal) and external (e.g., environmental) factors. Among external factors, occupational stress has been extensively studied and identified as a major factor associated with burnout [2,7,8,20]. According to the effort-reward imbalance (ERI) theory proposed by Siegrist et al., the ERI, known as the major scale to measure occupational stress, is mainly derived from the imbalance of effort and reward [21]. In addition, some reviews and empirical studies have proposed a protective value of perceived organizational support (POS) on burnout [22–24]. Among internal factors, drawing from positive psychology and positive organizational behavior (POB) [25], psychological capital (PsyCap) is receiving the increasing recognition of the value of improving employees’ physical and psychological health. PsyCap is a higher-order core construct that consists of the four state-like psychological resource capacities of self-efficacy, hope, optimism, and resilience, which can all be measured, developed, and effectively managed [26,27]. PsyCap has been reported as a positive resource for improving employees’ job performance [28], job satisfaction [29], and for combating employees’ stress, turnover and depressive symptoms [30,31]. In addition, PsyCap is identified as a mediator in the relationship between work-family conflict and burnout among Chinese female nurses [6]. Although occupational stress, ERI, POS, and PsyCap are found to be separately related with burnout among various occupational groups [2,7,6,24], to our knowledge, little research has studied the external and internal factors that contribute to burnout among Chinese iron and steel workers.

In the present study, we investigated the burnout situation of Chinese iron and steel frontline production workers in the Anshan Iron-Steel Complex in Anshan, China. The aim of this study was to explore external and internal factors associated with burnout among iron and steel workers in China. We selected ERI (as occupational stress), POS, and PsyCap as external and internal factors because they have been applied widely in work-related burnout, and are relatively easy to measure [32]. Occupational stress and Pos are external factors, and ERI is used for measurement of occupational stress. PsyCap is an internal factor. This study aimed to test the following hypotheses: 1) effort-reward imbalance is positively associated with burnout level; and 2) POS and PsyCap are negatively associated with burnout level among Chinese iron and steel workers. Our findings may be used to develop potential interventions to reduce burnout among iron and steel workers.

## Methods

### Study Design and Study Sample

In this cross-sectional study, we assessed burnout situation of iron and steel frontline workers in the Anshan Iron-Steel Complex, the largest integrated iron-steel company in Northeastern China. Only full-time workers who worked for at least one year in the Anshan Iron-Steel Complex were included in the study. The participants all have been involved in production and direct exposure to heavy manual labor or factory hazards, who are from steel melting section, rolling mill section, quality control department etc. Workers from administrative and technical areas were excluded from the study, as they are not exposed to the above hazards under normal circumstances. We distributed self-administered questionnaires to 1600 registered production workers. All participants were totally voluntary and anonymous, and were orally informed about the contents and aims of the questionnaire by researchers. There were no penalties or rewards for participations in the study. Researchers were ready to answer to all of their questions. Workers primarily from the iron, steel, coke, and steel casting plant received and completed questionnaires during working time.

Finally, 1300 complete questionnaires were returned to the researchers (response rate: 81.3%). 300 questionnaires were excluded from the study due to incompleteness or failure to return. The study was approved by the Committee on Human Experimentation of Harbin Medical University, and written informs consents was given by all of the participants. The study procedures were in accordance with the ethical standards.

### Measurement of Sociodemographic Characteristics

Age, gender, marital status, educational level, and monthly income were collected in this study. Marital status was categorized as single, married/cohabiting, and divorced/widow/separated. Education level was categorized as senior school or under, junior college, and undergraduate or above. Monthly income was categorized as 3000 yuan, 3001–4000 yuan, and > 4000 yuan. In the process of hierarchical regression analysis, the “undergraduate or above”, “female”, “Divorced/Widow/Separated” and “>4000” groups were defined as a reference group for the education level, sex, marital status and income, respectively.

### Measurement of Burnout

Burnout was measured using the Chinese version of the Maslach Burnout Inventory -General Survey (MBI-GS) [33], as previously reported [2,6,34]. The Chinese version of MBI-GS consisted of three dimensions including 16 items: emotional exhaustion (five items), cynicism (four items), and professional efficacy (seven items). All the items were scored on a Likert scale from 0 (never) to 6 (every day). High emotional exhaustion and cynicism dimensions combined with low professional efficacy dimension indicated a high level of burnout. In this study, the Cronbach’s α value for the total 16 items was 0.863. The Cronbach’s α values for emotional exhaustion, cynicism, and professional efficacy were 0.935, 0.903, and 0.889, respectively.

### Measurement of Occupational Stress

Occupational stress was evaluated using the effort-reward imbalance (ERI) model, which was built upon a reciprocal relationship between efforts and rewards [35]. Efforts represent job demands and/or obligations that are imposed on the employee. Rewards consist of money, esteem, and job security/career opportunities. Work characterized by both high efforts and low rewards represents a reciprocity deficit between efforts and rewards. The imbalance between efforts and rewards may cause sustained occupational stress [35].

The Chinese version of the ERI scale was translated and provided by Yang and Li [36]. The original version of the ERI scale consists of three subscales: effort (6 items), reward (11 items), and overcommitment (6 items). Overcommitment, defined as a motivational personality characteristic, was excluded because: (a) occupational stress was considered as an external (or situational) factor in our study; (b) overcommitment is a moderator, but not a direct factor, between ERI and occupational stress by intensifying ERI [37,38]. The effort and reward subscales were scored on a 5-point Likert scale (ranging from ‘not distressed’ to ‘very distressed’) after answering to an initial question on whether the item applied (yes or no). From the ERI theory, occupational stress could be expressed by the effort/reward ratio (ERR) [35,37]. The ERR was calculated by putting the effort score in the numerator and the reward score in the denominator. The reward score was multiplied by a coefficient (0.5454) to adjust the unequal number of items in the two scales. A score of ERR greater than 1 indicates a high amount of effort spent that is not met with adequate reward. In this study, the Cronbach’s α value for total 17 items was 0.552. The Cronbach’s α values for effort and reward subscales were 0.866 and 0.900, respectively.

### Measurement of POS

POS was assessed using the 9-item version of the Survey of Perceived Organizational Support (SPOS) [39], which has been extensively applied and validated among Chinese occupational groups [31,40]. The scale consisted of statements mainly concerning whether the organization appreciated employees’ contributions and treated them favorably or unfavorably in different circumstances. Each item was scored on a 7-point Likert scale ranging from 1 (strongly disagree) to 7 (strongly agree). The total score ranged from 7 to 63 scores, and higher score indicated higher level of POS. The Cronbach’s α value for the POS scale was 0.878 in this study.

### Measurement of PsyCap

PsyCap was assessed using the Chinese version of the 24-item Psychological Capital Questionnaire (PCQ) [6,31]. PCQ has adequate reliability and construct validity across multiple samples [6,28,30,31]. PCQ comprises of four subscales containing six items for each subscale (self-efficacy, hope, resilience, and optimism). Each item was scored on a six-point Likert-type scale with categories ranging from 1 (strongly disagree) to 6 (strongly agree). The total score ranged from 24 to 144 scores, and higher score indicated higher level of PsyCap. The Cronbach’s α value for total 24 items was 0.912. The Cronbach’s α values for self-efficacy, hope, resilience and optimism were 0.841, 0.852, 0.748, and 0.636, respectively.

### Statistical Analysis

Analyses were performed using SPSS 13.0. Independent sample t-test or one way analysis of variance (ANOVA) was used to analyze the difference in burnout among different groups, followed by least-significant-difference (LSD) tests. Pearson’s correlation tests were used to examine association between variables. Hierarchical regression analysis was used to explore the effects of occupational stress, POS and PsyCap on burnout with adjustment for demographic and working variables. Univariate analysis was used to evaluate the association between burnout scores and the demographic and working variables. All the variables including those that were not significantly associated with burnout were entered into the step 1 of the hierarchical regression analysis. Data including R^2^, adjusted R^2^ (Adj.R^2^), R^2^-changes, F, standardization regression coefficients (β) and p values for each step were included in the regression model. Moreover, tolerance and variance inflation factor were used to check for multicollinearity. Probability values < 0.05 were considered statistically significant.

## Results

### MBI-GS Scores in Iron and Steel Workers

Mean MBI-GS scores were 13.11±8.06 for emotional exhaustion, 6.64±6.44 for cynicism, and 28.96±10.39 for professional efficacy. Table 1 summarizes MBI-GS scores in iron and steel workers according to sociodemographic features. Mean MBI-GS scores of emotional exhaustion, cynicism, and professional efficacy were significantly different among workers with different ages (p<0.05). Mean MBI-GS scores of emotional exhaustion and cynicism, but not professional efficacy, were significantly different in workers with different education levels (p<0.05). Mean MBI-GS scores of emotional exhaustion, cynicism, and professional efficacy were not significantly different in workers with different gender, marital status, or monthly income (p>0.05).

**Table 1:** Mean MBI-GS scores of iron and steel worker according to sociodemographic feature.

Variables	N (%)	Emotional exhaustion	Cynicism	Professional efficacy
		Mean (SD)	Mean (SD)	Mean (SD)
Age(years)		P = 0.019	P = 0.003	P = 0.003
≤30	155(11.9)	12.40(7.99)^a^	5.71(6.01)^a^	31.14(11.22)^a^
31–40	369(28.4)	14.10(7.89)^b^	7.13(6.12)^b^	28.68(9.79)^b^ ^/^ ^c^
41–50	618(47.5)	12.99(8.32)^a^	6.92(6.86)^b^	29.11(10.16)^b^
≥51	158(12.2)	11.99(7.23)^a^	5.27(5.61)^a^	26.85(11.37)^c^
Gender		P = 0.262	P = 0.348	P = 0.151
Male	1233(94.8)	13.17(8.11)	6.67(6.50)	29.05(10.36)
Female	67(5.2)	12.04(7.05)	6.05(5.16)	27.18(10.92)
Marital status		P = 0.627	P = 0.054	P = 0.331
Single	93(7.2)	13.07(7.26)	7.57(6.29)	27.75(10.07)
Married/Cohabitation	1098(84.5)	13.19(8.15)	6.68(6.49)	29.14(10.30)
Divorced/Widow/Separated	109(8.4)	12.41(7.77)	5.43(5.93)	28.16(11.46)
Education level		P = 0.001	P = 0.001	P = 0.295
senior school or under	740(56.9)	12.32(8.04)^a^	6.09(6.22)^a^	28.69(10.82)
junior college	416(32.0)	14.26(7.83)^b^	7.25(6.60)^b^	28.99(9.60)
undergraduate or above	144(11.1)	13.87(8.36)^b^	7.69(6.83)^b^	30.17(10.28)
Monthly income(yuan)		P = 0.289	P = 0.596	P = 0.112
≤3000	995(76.5)	12.92(8.03)	6.72(6.38)	28.76(10.36)
3001–4000	217(16.7)	13.63(8.08)	6.49(6.69)	28.92(10.68)
>4000	88(6.8)	13.98(8.26)	6.04(6.53)	31.18(9.80)

^a,b,c^ Calculated by least-significant-difference (LSD), mean scores for the three dimensions of burnout with different superscripts differ significantly at the p<0.05 level.

### Correlations among Occupational Stress, POS, PsyCap and Burnout

Table 2 summarizes correlations among age, occupational stress, POS, PsyCap, and burnout, using Pearson correlation analysis. ERR was positively related with emotional exhaustion, cynicism. POS and PsyCap were positively correlated with each other. Both POS and PsyCap were negatively associated with emotional exhaustion, cynicism, effort, and ERR, but positively correlated with professional efficacy and reward. In addition, PsyCap was positively associated with age.

**Table 2:** Means, standard deviations (SD), range, and correlations of continuous variables.

Variables	Mean(SD)	Range	1	2	3	4	5	6	7	8	9
**1.Age**	41.86(8.24)	22–60	1								
**2.Emotional Exhaustion**	13.11(8.06)	0–30	0.013	1							
**3.Cynicism**	6.64(6.44)	0–24	0.029	0.634**	1						
**4.Professional Efficacy**	28.96(10.39)	0–42	-0.069*	0.094**	-0.047	1					
**5.Effort**	15.60(6.04)	6–30	-0.017	0.583**	0.447**	0.038	1				
**6.Reward**	42.96(9.91)	11–55	0.038	-0.408**	-0.478**	0.138**	-0.638**	1			
**7.ERR**	0.77(0.51)	0.2–5	-0.033	0.516**	0.501**	-0.034	0.829**	-0.841**	1		
**8.POS**	41.98(10.75)	9–63	-0.010	-0.358**	-0.471**	0.187**	-0.353**	0.514**	-0.426**	1	
**9.PsyCap**	103.32(16.37)	29–144	0.055*	-0.204**	-0.357**	0.308**	-0.186**	0.374**	-0.265**	0.538**	1

* p<0.05,

** p<0.01

Note: ERR = effort/reward ratio; POS = perceived organizational support; PsyCap = psychological capital

### Hierarchical Regression Analyses

The external and internal factors associated with the each dimension of burnout after adjusting for demographic and working variables were shown in Table 3. ERR and POS together accounted for an additional 30.8% variance to the prediction of emotional exhaustion in step 2, but PsyCap did not account for an additional variance of emotional exhaustion in step 3 (R^2^-changes = 0). ERR (β = 0.491, P < 0.001) and POS (β = -0.144, P < 0.001) represented significantly individual predictive values. As for the cynicism, ERR and POS together accounted for an additional 30.3% variance to the prediction of cynicism in step 2, and PsyCap accounted for an additional 1.2% variance of cynicism in final step. ERR (β = 0.334, P < 0.001), POS (β = -0.252, P < 0.001) and PsyCap (β = -0.131, P < 0.001) also represented significantly individual predictive values. With regard to professional efficacy, ERR, POS and PsyCap accounted for 10% incremental variance to professional efficacy. The test of R^2^-change was significant (F change (3, 1264) = 48.313, P < 0.001), suggesting ERR, POS and PsyCap together to be significantly predictive of professional efficacy, however neither ERR (β = 0.053, P = 0.073) nor POS (β = 0.046, P = 0.168) were significant independent individual predictors of professional efficacy when PsyCap (β = 0.302, P < 0.001) was added in step 3. Finally, Tolerance and variance inflation did not indicate a multicollinearity problem.

**Table 3:** Hierarchical regression analysis for exploring the external and internal factors on the three dimensions of burnout.

Variables	Emotional exhaustion			Cynicism			Professional Efficacy		
	Step 1(β)	Step 2(β)	Step 3(β)	Step 1(β)	Step 2(β)	Step 3(β)	Step 1(β)	Step 2(β)	Step 3(β)
**Control variables**									
Age	-0.027	-0.028	-0.028	-0.001	-0.006	0.000	-0.079**	-0.074**	-0.090**
Gender	-0.032	-0.022	-0.022	-0.026	-0.024	-0.024	-0.040	-0.035	-0.036
Education level 1	-0.089	0.058	0.059	-0.129**	0.013	0.005	-0.057	-0.087	-0.068
Education level 2	0.028	0.075*	0.076	-0.039	0.004	-0.006	-0.044	-0.051	-0.029
Marital status 1	-0.017	-0.044	-0.044	0.055	0.024	0.023	-0.026	-0.015	-0.012
Marital status 2	0.015	0.023	-0.022	0.058	0.026	0.023	0.023	0.026	0.033
Income1	-0.030	-0.076	-0.077	0.073	0.006	0.012	-0.086	-0.055	-0.068
Income2	-0.009	-0.035	-0.035	0.034	0.001	0.003	-0.076	-0.063	-0.068
**External factors**									
ERR^a^		0.488***	0.489***		0.340***	0.333***		0.027	0.041
POS		-0.147***	-0.156***		-0.319***	-0.253***		0.202***	0.044
**Internal factors**									
PsyCap			0.017			-0.127***			0.303***
**F**	2.649**	62.423***	56.759***	2.470*	59.288***	56.757***	2.301*	6.712***	15.070***
**R** ^**2**^	0.016	0.326	0.326	0.015	0.315	0.326	0.014	0.049	0.114
**Adj.R** ^**2**^	0.010	0.321	0.321	0.009	0.310	0.321	0.008	0.042	0.10
**R** ^**2**^ **-changes**	0.016	0.310	0.000	0.015	0.300	0.011	0.014	0.035	0.065

* p<0.05,

** p<0.01,

*** p<0.0001

Note: ERR = effort/reward ratio; POS = perceived organizational support; PsyCap = psychological capital; Adj.R^2^ = adjusted R^2^; Education level 1 = Senior school or under vs. undergraduate or above, Education level 2 = junior college vs. undergraduate or above; Marital status 1 = Single vs. Divorced/Widow/Separated; Marital status 2 = Married/Cohabitation vs. Divorced/Widow/Separated; Income1 = ≤3000 vs. >4000; Income2 = 3001–4000 vs. >4000.

^a^ A logarithmic transformation of ERR was performed to correct for Skewness and Kurtosis

## Discussion

### Burnout Level

In the present study, to obtain a large sample of subjects, we selected iron and steel workers in the Anshan iron-steel complex, the largest integrated iron-steel company in Northeast China. Of 1600 participants, 1300 participants returned complete questionnaires (response rate of 81.3%), and thus we obtained a large sample size that provided a good representation of our study population and allowed for possible generalization of our conclusions.

The steel frontline workers who are exposed to a wide variety of toxic substances work under very stressful working conditions, and thus are more associated with psychological and behavioral problems. They are more likely to have constant negative emotions such as irritability, rage, and anxiety, and less enthusiasm for work. In addition, since they commonly lack knowledge about occupational health, and rarely realize these occupation-associated negative emotions, they are not treated promptly. Therefore, the iron and steel workers have a high level of burnout [9]. In the present study, we found that the mean MBI-GS scores of burnout of iron and steel frontline workers (mean scores divided by items number) were 2.62 for emotional exhaustion, 1.66 for cynicism, and 4.14 for professional efficacy. The level of emotional exhaustion and cynicism in iron and steel frontline workers is similar to that in other occupations with a relatively high level of burnout, such as nurses (n = 1478, emotional exhaustion = 2.35, cynicism = 1.78) [2], doctors (n = 1202, emotional exhaustion = 2.29, cynicism = 1.73) [41], and teachers (n = 558, emotional exhaustion = 2.04, cynicism = 1.14) [42], and even higher than that of police officers (n = 221, emotional exhaustion = 1.38, cynicism = 1.50) [43], using the same measurement method (MBI-GS). Therefore, iron and steel frontline workers are at relative risk for emotional exhaustion and cynicism. However, the level of professional efficacy in iron and steel frontline workers was higher than that in nurses (n = 1478, professional efficacy = 3.33) [2], doctors (n = 1202, professional efficacy = 3.44) [41], and teachers (n = 558, professional efficacy = 3.86) [42], and similar to that in police officers (n = 221, professional efficacy = 4.72) [43]. The higher level of professional efficacy in iron and steel worker may be because their job requires simple technique, skill, and knowledge. In contrast, police officers, nurses, doctors and teachers need to constantly adopt and refine some techniques, skills and knowledge, and thus the difficulty of fulfilling their tasks, to some extent, may reduce the level of professional efficacy.

### Association of Burnout Levels with Sociodemographic Characteristics in Iron and Steel Workers

In the present study, we found that age and education level was associated with burnout levels in iron and steel workers. Workers with the age between 31 and 50 years old had a higher level of emotional exhaustion and cynicism. This finding is consistent with a previous study showing that the association age was positively associated with burnout in middle-aged working population in Finland [44]. In addition, we found that young workers (≤30 years old) had the higher level of professional efficacy. The higher level of professional efficacy in young workers may be because young workers in the beginning of their career devoted their enthusiasm and energy to their work in order to achieve their initial goals. The high level of self-confidence and involvement may increase the feeling of competence and successful achievement regarding their jobs. Furthermore, higher education was also found to be associated with higher levels of emotional exhaustion and cynicism. Iron and steel workers with higher education levels who have higher levels of emotional exhaustion and cynicisms are shunning factory work [16]. Similar findings also occurred in Chinese coal workers [45].

It has been reported that men often score higher on emotional exhaustion and cynicism than women, and women are more likely than men to use social support to cope with burnout [46,47]. However, in the present study, we found that there were no significant differences in burnout between males and females. Since females account for a very small portion of our sample (5.2%), lack of sufficient sample size may cause no significant differences in burnout between males and females in our study.

### Internal and External Factors Associated with Burnout

Among internal and external factors, we found that ERI was the major factor associated with burnout subscales of emotional exhaustion and cynicism, which was consistent with previous studies [2,7,8]. Because of the highly harsh work environment, iron and steel workers not only were exposed to chemical, physical, and biological hazards, and had a high rate of work-associated diseases and absenteeism. However, they were lack of adequate reward (income) and received much less attention from government and researchers [16]. Although the average income of the iron and steel workers in Anshan Iron and Steel Group Corporation is higher than in other small and medium sized steel and iron companies, it is still relatively low in the society. Since the high effort was not met with appropriate reward, iron and steel workers may feel emotionally overwhelmed and exhausted by work (emotional exhaustion) and doubt the value of their own work or their contributions to anything (cynicism) [48]. With the rapid economic growth of China, the work environment has been improved gradually and the workers’ income has substantially increased [48]. The effort/reward ratio will expected to be better, and the burnout level will be likely reduced in the future.

POS has been considered as an externally effective resource that predicts a wide range of positive work attitudes and outcomes [22–24]. In the present study, we found that POS had a negative effect on burnout subscales of emotional exhaustion and cynicism, suggesting that POS provided encourage, fulfillment, and help for workers, paid attention to their well-being and contributions, and promoted identification and willingness with their own work [22]. In China, Iron and steel workers received less support and attention from society and government. Our findings suggest that if the organization, (e.g. the Anshan iron-steel complex) valued the workers’ contributions and accomplishments and provided appropriate help and support, they could be fully engaged and dedicated to their work.

As an important internal and personal resource, PsyCap goes beyond human capital, social capital, and financial capital in obtaining and sustaining the competitive advantage in today’s organization [26]. It has been suggested that PsyCap decreases employees’ stress, turnover, and depressive symptoms, and has the potential for improving job performance and job satisfaction [28–31]. However, the relationship between PsyCap and burnout has not been well investigated, and is only reported among Chinese nurses [6,49]. In the present study, we found that PsyCap was a predictor of low cynicism and high professional efficacy, which agrees with previous reports [6,49]. Interestingly, PsyCap was the most salient factor related to professional efficacy among the subscales of burnout. This is possibly because professional efficacy is a cognition/appraisal-oriented construct that is described as a feeling of competence and successful achievement in one’s work with people [2], whereas emotional exhaustion and cynicism, to some extent, are considered as emotion-oriented constructs. Effective measurement and management of PsyCap can improve confidence and belief in workers’ ability to achieve goals and fulfill challenging tasks, and thus can increase and sustain the enterprise competitiveness [25–27]. Our finding suggests that PsyCap is an important internal resource for enhancing workers’ professional efficacy.

### Implications

Our study provides several practical implications. First, in order to alleviate the imbalance between effort and reward and decrease burnout among iron and steel workers, further studies are required to develop a targeted intervention to keep effort-reward balance (such as work hours, fair work allocation, promotion, stability, respect, support, and incomes), using the ERI model. Second, in addition to alleviate effort-reward imbalance, enterprise managers should improve POS in workers by helping them with career planning, improving job autonomy, and providing supervisor support [21,31]. Third, to improve enterprise competitiveness and job performance, steel enterprises mainly focus on the human capital and financial capital, but ignore the workers’ internal psychological resource. Several studies have shown that interventions designed to improve PsyCap including self-efficacy, hope, resilience and optimism are effective to reduce occupational burnout [6,31,50,51,52]. Therefore, effective strategies should be implemented to improve PsyCap and relieve burnout in Chinese iron and steel workers. Finally but most importantly, we found that internal and external factors had different effects on the three subscales of burnout. Therefore, it is important for researchers to build an integrated model including both internal and external to decrease burnout in iron and steel workers.

### Limitations

The present study has some limitations. First, given the cross-sectional design, we were unable to draw any causal conclusion about the association between burnout and internal or external factors. A future longitudinal study is required to confirm our findings. Second, this study only included iron and steel workers from the Anshan iron-steel complex in northeast China. It is possible that some factors specific for the Anshan iron-steel complex may not be generalized to other complex. Third, this study only included full-time workers, not precarious workers such as temporary workers, contract workers, and part-time workers in this study because the proportion of precarious workers is low in the complex. We cannot obtain a large sample size for accurate analysis. Further studies in multiple iron-steel complexes may be performed to determine the internal and external factors associated with burnout in precarious workers. Fourth, this study preliminarily investigated association of ERI, POS and PsyCap with burnout in iron and steel worker, other internal and external factors related to burnout should be further explored in the future. Fourth, since all data were obtained using self-administered questions, there was a possibility of recall or reporting bias. The frequency of the burnout symptoms was retrospectively reported, and thus the recalled information was dependent on the memory of the workers, which can be imperfect and inaccurate. The report results may also be influenced by the general beliefs on burnout from semantic memory. It is hypothesized that individuals with more burnout would recall more frequent symptoms than those with less burnout when reporting their questionnaire [53]. Finally, in the present study, we evaluated occupational stress by ERI. However, other occupational stress such as job control, role conflict, role ambiguity, and person-environment fit was not included in the present study.

## Conclusions

In the present study, we found that Chinese iron and steel workers has a high level of burnout. Burnout might be associated with internal and external factors, including ERI, POS and PsyCap. ERI was found to be the most robust factor of emotional exhaustion and cynicism. These internal and external factors had different effects on the three subscales of burnout. Our findings highlight the need for managers to be aware of the risk of burnout among iron and steel workers. An integrated model including both internal and external factors should be developed to reduce the level of ERI, and improve POS and workers’ PsyCap, thereby alleviating the level of burnout among iron and steel workers.
